# The Status of Newborn Hearing Screening in Japan: Past, Present, and the Future

**DOI:** 10.7759/cureus.28858

**Published:** 2022-09-06

**Authors:** Jason L Hollowell, Akira Takagi

**Affiliations:** 1 Hearing Impairment Identification and Intervention, Hearing and Language Center, Shizuoka General Hospital, Shizuoka, JPN; 2 Global Studies, Musashi University, Tokyo, JPN; 3 Otolaryngology, Hearing and Language Center, Shizuoka General Hospital, Shizuoka, JPN

**Keywords:** early intervention, hearing impairment, screening, follow-up testing, tracking, newborn hearing screening

## Abstract

This review article summarizes the chronological history of newborn hearing screening (NHS) implementation in Japan. Beginning with experimental pilot programs implemented in the early 2000s, efforts have been made to establish NHS throughout the country. The results of and responses to these pilot programs are introduced, analyzed, and discussed. Data reported annually, from 2014, by the Japanese Ministry of Health, Labour, and Welfare (MHLW), introduce the overall progress achieved in NHS throughout the country. The most recently published MHLW report, from 2019, cites a screening rate of 90.8%. Analysis of the data from these reports, however, suggests that while clear progress has been achieved, “known-screening” rates are lower than the “surveyed” rates cited. Published NHS program data from three pilot programs as well as publicly available data from one prefecture and unpublished data from an additional prefecture are analyzed and compared to the national figures. Hearing impairment occurrence frequency for newborns in Japan, calculated from two large data sets, reveals an average occurrence rate of 0.14% or one per every 1,400 births. Progress is observed in terms of an expanding coverage rate for NHS in Japan. Work remains, however, to achieve a screening rate of 95% or higher. Additionally, a protocol for ensuring quality standards for NHS is recommended. Data collected and analyzed for this review may inform planned efforts to introduce more efficient digitized NHS program management systems in Japan as well as in other countries where NHS program improvement efforts continue. Such systems may serve to enable effective monitoring of pre-determined screening program protocols and thus may make a shift from a 1-3-6 to 1-2-3 protocol more feasible.

## Introduction and background

The 1995 World Health Organization (WHO) Forty-Eighth World Health Assembly Resolution, 48.9, urges member states to include early detection programs for hearing impairment within primary healthcare frameworks [1]. A subsequent WHO Informal Consultation, in 2000, recommended a “policy of universal neonatal screening be adopted in all countries and communities with available rehabilitation services” [2]. During this time, research documenting the practicality and benefits of newborn hearing screening (NHS) gradually accumulated [3-5].

The new recommended benchmark for early detection and intervention for children with hearing impairment, published by the Joint Committee on Infant Hearing, is for localities to strive to meet a 1-2-3 timeline (testing by one month, diagnosis by two months, and intervention by three months of age) after they have achieved the previously recommended 1-3-6 timeline published in 1994 [6,7]. One of the main goals behind these goals is to ensure maximal levels of language acquisition for children with hearing impairment. Advancements in the field of cochlear implantation have made the use of this early intervention option more accessible at very early ages in addition to traditional amplification via the use of hearing aids or the use of visual modes for language acquisition [8].

This review attempts to determine the degree to which NHS programs in Japan meet or exceed the original 1-3-6 timeline, to identify factors inhibiting successful implementation, and to suggest pathways for achieving the new accelerated goals. This includes an evaluation of the primary factor, what percentage of newborns are screened by one month of age, as well as consideration of the percentage of referred newborns receiving follow-up testing and of those, what percentage are diagnosed with hearing impairment. When possible, false positive rates and positive predictive values are presented.

For an NHS program to successfully identify children with hearing impairment and thus subsequently enable timely and effective intervention, it must provide coverage for all newborns. As such, the initial fundamental criteria for evaluating the effectiveness of a program is the percentage of all newborns provided with a hearing screening test (i.e., the screening rate). National data, reported by the Ministry of Health, Labour, and Welfare, indicate that while progress has been made, work still remains to achieve a 95% or higher target screening rate [9]. Additionally, screening tests should have a high level of sensitivity and specificity. That is, testing should be sufficiently sensitive to identify hearing impairment when it exists, and also sufficiently specific to eliminate children who do not have a hearing impairment. However, the present review, which analyzed published and unpublished data, suggests that while national NHS rates are climbing, data enabling thorough evaluation of sensitivity and specificity are rarely recorded or available. Additionally, evaluation of screening programs, from the perspective of the 1-3-6 and 1-2-3 protocols remains problematic due to a paucity of information available about follow-up testing for children who receive a referral on their first test as well as about early intervention for children identified with hearing impairment.

## Review

Methodology

The analyses for this review consisted of four parts. First, we collected published materials and relevant information regarding pilot programs: In 2000, the Japanese Ministry of Health, Labour, and Welfare announced an NHS pilot program and called for prefectures to apply for funding and participate in the program. The island nation of Japan is comprised of 47 prefectures which are similar to States or Provinces. The Prefecture is the primary regional governmental organizational unit, and it is frequently at this level that initiatives, introduced and funded by the federal government, are introduced and implemented. Four prefectures initially, Okayama, Kanagawa, Tochigi, and Akita, are reported to have applied for and participated in the four-year pilot program [10]. A summary of three of these pilot programs is presented as a literature review. These programs are analyzed on their achieved screening rates, referral rates, and positive predictive values.

Second, we reviewed the literature available on reactions to the pilot programs. Various publicly presented views on the efficacy of NHS, its costs, and the role of screening as a means to enable early intervention for children with hearing impairment are presented. Additionally, the stance of the Japanese government, in regard to conducting NHS and funding screening programs, is introduced. These are summarized in the section titled Challenges for NHS in Japan.

Third, we analyzed two ongoing prefectural NHS programs, the Tottori Prefecture NHS program, and the Shizuoka Prefecture NHS program. We derived indices such as the achieved screening rate, referral rate, false positive rate, and positive predictive value for each prefecture.

Finally, we analyzed national NHS status data provided in annual NHS reports and birth statistics published by the Japanese Ministry of Health, Labour, and Welfare (section 1.6). The national reports are analyzed on the achieved screening rates and referral rates. Additionally, for all data sets, when possible, hearing impairment occurrence frequency rates were calculated, analyzed, and compared.

Pilot programs in the early 2000s

The Japanese federally funded NHS pilot programs were conducted in various localities, for a period of four years each, beginning in 2000. The funding program ceased in March 2004 with ongoing programs completed in 2006 [11]. Subsequently, the Japanese government urged prefectures to conduct NHS using an alternative, already existing, government funding scheme. NHS, in these programs, was conducted while the mother and child were still receiving post-birth care at birthing facilities which include public hospitals and private hospitals and clinics. Outcome reports, for each of the three prefectural programs introduced, include data summarizing the achieved screening rates.

It should be noted that each summary includes observations about some of the difficulties associated with administering an NHS program. One issue is the difficulty in counting total screening tests per total annual prefectural births due to the common practice in Japan of “Satogaeri Bunben” (returning home to give birth). It is commonly the practice for an expecting mother to return to her home prior to giving birth and to stay there for some time after the birth for support from the family [12]. In these cases, the newborn may be screened where it was born but registered in the prefecture where the mother resides. This not only makes calculating screening rates difficult as the birth is registered in a different prefecture, but it also complicates follow-up efforts for children who receive screening referrals.

Okayama Prefecture

Fukushima et al. [12] report on Okayama Prefecture’s participation in the NHS pilot program which began in 2001. Forty-four hospitals were enrolled in the pilot program and over the course of the four years, 49,839 infant births were recorded in these facilities. 47,346 infants are reported to have been screened, representing 94.9% of the children born in the participating facilities during the pilot program. During the same four years, however, 71,198 childbirths were reported for the entirety of Okayama Prefecture. As such, the “known-screening” rate, from the perspective of this pilot program, is calculated to be 66.5% (47,346/71,198). Of the children screened, 0.52% received referrals. Approximately 1,270 of the annual 19,000 births are reported to have been “Satogaeri” return-home births [12]. Despite the various challenges, the percentage of hearing-impaired children referred before the age of six months increased from almost none in 1995 to 30% in 1998, and later, as some hospitals introduced NHS, to 70% by the end of the pilot program in 2005. Fukushima et al. report 248 infants, over the course of the four-year program, as having been identified via screening for follow-up testing. Of the 248, 140 were identified with possible unilateral hearing impairment and 108 with possible bilateral hearing impairment. Only the bilateral 108 cases are reported with 40 having been identified to have a hearing impairment, representing a positive predictive value of 37% for this subgroup of 108 children. Using the bilateral hearing impairment data, a hearing impairment occurrence frequency of 0.08% is cited. The basic details of the Okayama Prefecture program are summarized in Table 1.

**Table 1:** Summary of basic details of the Okayama Prefecture NHS pilot program.

Time Period	# Participating Facility Reported Births	# Screened	Participating Facility Screening Rate	# Total recorded births	Overall “Known-Screening” Rate	Referral Rate	PPV	HI Rate
2001~2005	49,839	47,346	94.90%	71,198	66.50%	0.52%	37%	0.08%

Akita Prefecture

Akita Prefecture participation in the NHS pilot program began in 2001. Basic details of the program are reported by Mizuno et al. [13] in a 13- year screening summary from the perspective of the Akita University Hospital. While exact numbers are not provided, a graph comparing total screening numbers to the number of births per year shows that in 2004, the final year of participation in the pilot program, roughly 4,500 screenings were conducted while in the same year roughly 8,000 childbirths were reported. As such, the “known screening” rate for this year in Akita is calculated to be approximately 56.3% (4,500/8,000). Mizuno et al. [13] note, however, the difficulties associated with counting unique screenings per birth for the purpose of calculating the overall screening rate. This difficulty arises from the fact that multiple “confirmation” screening tests and tests administered to “Satogaeri” return-home children are included in the total and thus inflate the numbers. In their data, for example, in 2013 roughly 7,000 screenings are recorded for approximately 6,500 births. Nevertheless, Mizuno et al. [13] report Akita to have achieved a screening rate of 100% in 2012. Additionally, the 13-year screening summary provides information on the 90 individuals identified with hearing impairment. The 90 confirmed cases were from 142 follow-up tests conducted out of a total of 148 referrals. This reveals a positive predictive value of 63.4%. However, details about the number of screenings performed per birth are not provided. As such, hearing impairment occurrence frequency rates could not be calculated. The basic details of the Akita Prefecture program are summarized in Table 2.

**Table 2:** Summary of basic details of the Akita Prefecture NHS pilot program.

Year	# Participating Facility Reported Screening Count	# Total Prefectural Births	Overall “Known-Screening” Rate	Referral Rate	PPV	HI Rate
2004	4,500	8,000	56.30%	---	63%	---

Tochigi Prefecture

Tochigi Prefecture participation in the NHS pilot program began in April 2002 and finished in March 2006. Over the course of the four-year program 6,198 of the 71,677 children born in the prefecture during the same period, are reported to have been screened indicating an overall known screening rate of 8.6% [14]. The newborns screened were separated into high-risk and low-risk groups and referral rates, follow-up testing rates, and hearing impairment diagnosis rates were calculated for each group separately. For the four years of the pilot program, 44 of the 6,198 children screened were identified to have hearing impairment of 35 dB or greater representing 0.7% of the screened population [14]. The hearing impairment occurrence frequency for the low-risk group (4,683) was 0.13% and for the high-risk group (1,515) 1.58%. As shown in Table 3, assuming the proportion of low risk to high-risk births can be extrapolated to the entire birth cohort of 71,677 newborns, approximately 347 newborns can be estimated to have been born with some degree of hearing impairment during this time period. Based upon their analysis of the four-year program, Fukami et al. conclude that a universal newborn hearing “mass screening” program, implemented without the proper follow-up and habilitation services in place, would be meaningless and they express hope for a government-led implementation of post-diagnosis intervention services [14].

**Table 3:** Estimated number of children born with hearing impairment between 2002 and 2008 in Tochigi Prefecture based upon data reported by Fukami et al. HI = hearing impairment

Risk Group	# Screened	# Diagnosed with HI	HI Rate	Percentage of total screened	Proportion * total births	HI #s Estimate
Low-Risk	4,683	6	0.13%	75.6%	54,157	69
High-Risk	1,515	24	1.58%	24.4%	17,520	278
Total	6,198	30	0.48%	100%	71,677	347

Screening rates, both reported and “known,” for the three pilot programs summarized above differ greatly ranging, in the case of the “known rate,” from 8.6% in Tochigi Prefecture to 66.5% in Okayama Prefecture. The vast difference in screening rates, viewed at the prefectural level, is due to the fact that, while conducted as prefectural programs, only a subset of all of the birthing facilities in each prefecture participated in the program. Additionally, for participating facilities, the likelihood of a family opting out of an optional test may have been higher due to factors such as lack of information about early detection of hearing loss in infants, cost of the test, and apprehension about testing due to lack of sufficient information about the testing process.

Challenges for NHS in Japan

As the government-funded pilot programs accumulated preliminary data providing information about the efficacy of NHS, criticisms of the programs also arose. Some such critiques, such as those referenced previously by Fukami et al., focused on concerns about the need for the establishment of follow-up programs for children who received referrals as well as for those subsequently identified with hearing impairment. Others focused on the stress caused by the NHS programs. For example, at a symposium held in Tokyo in 2003, the Director of the Head Office of the Japanese Federation of the Deaf, denounced the screening programs on the basis of the anxiety they caused and called for their immediate discontinuation [15].

A 2005 literature analysis conducted by Okubo et al. [16] concluded that “the effectiveness of UNHS in Japan is still equivocal…” based upon a review and analysis of eleven research articles, nine from which only screening accuracy information was obtained with the remaining two also providing information about screening effectiveness. From the nine articles reporting on screening accuracy, the authors note a wide range in PPV values from 0% to 50% with referral rates ranging from 0.2% to 1.6%. Their two-point critique of the studies evaluated centered upon the fact that best practice standards were not reported in any of the studies and that distinction between low-risk and high-risk newborns was rare. The two remaining articles surveyed, which dealt with screening efficacy, were identified, by the authors, to have three limitations.

First, they identify a sampling bias in studies showing evidence of the benefits of early intervention with subjects all coming from one private institution and note that Japanese public schools for the deaf “play an important role in early intervention” [16]. Given the paucity of efficacy studies at the time, it is impossible to determine the degree or direction, positive or negative, of such a skewing factor.

The second critique in the Okubo et al. paper focuses on an identified lack of control over the length of intervention and difficulty in determining if the documented differences in IQ were affected by the timing of the start of intervention or its duration [16]. However, the two studies being evaluated used the Wechsler Preschool & Primary Scale of Intelligence (WPPSI) and the Wechsler Intelligence Scale for Children Third Edition (WISC III) to evaluate participants based on verbal IQ and performance IQ. Investigating the degree of attainment of age-appropriate auditory language ability necessitates the use of standardized measures capable of providing insights into developmental achievement. Research focused on the effect of duration of intervention, in these contexts, would have provided additional insight into developmental trajectories. It is, however, unclear whether its absence detracts from observations focused upon age-appropriate ability when investigating the efficacy of timing of intervention commencement.

The third critique centered on the fact that both studies focused solely on auditory-oral intervention programs. Early intervention programs utilizing the visual mode would shed further light on the role of intervention timing. Such additional information would further elucidate the impact of NHS in more clearly defined cohorts. How the absence of such information potentially confounds the outcomes observed in the stated population of children, who were participating in auditory-oral intervention programs, is not explained.

Newly starting government-funded NHS pilot programs ceased in 2006; however, the Japanese government continued to encourage prefectures to implement NHS and in 2007 issued a statement requesting all prefectures and metropolitan districts to offer NHS to all newborns [17]. The document explained that funding for these programs was being provided via a significantly increased funding program for “dealing with the declining birthrate.” The document also included a note indicating its purpose was “technical advice” under a local autonomy law from 1947 [17]. Several years later, in 2012, the Ministry of Health, Labour, and Welfare requested all screening results be recorded, by four months after birth, in the mother and child book, which is used by mothers to record various information related to the health of their child [18]. This proactive stance on NHS, from the Ministry of Health, Labour, and Welfare, was one factor influencing a request, in 2015, from a group of related healthcare entities including the Japan Association of Obstetricians and Gynecologists, the Japanese Society of Otorhinolaryngology-Head and Neck Surgery, among sixteen others [10]. The signatory groups requested funding from the national government for the implementation of NHS for all newborns. Both in response to this request and as a result of findings from a survey conducted in 2014, which found a very low rate of localities reimbursing for the cost of testing (109/1,741 or 6.3%), the Ministry of Health, Labour, and Welfare issued an additional notice in March of 2016 [19,20]. This notice restated that funding for NHS was, in fact, being provided within a significantly increased nationally funded program for dealing with the declining birthrate. Subsequent efforts, at the prefectural level, have been made to fortify NHS programs through cooperation between prefectural governmental agencies, hospitals and birthing clinics, and the individual locality offices responsible for providing reimbursement funding to testing hospitals and clinics. Two such programs are outlined in brief in the section below.

Prefectural NHS programs

Two prefectural programs were selected for this review. The first program, the Tottori prefecture program, was selected due to the wealth of information about its NHS program that is openly available via the Prefecture’s Internet homepage. The second program, the Shizuoka prefecture program, was selected because of the authors' affiliation with Shizuoka Prefectural General Hospital and, as such, access to data for aggregation and analysis. This set of unpublished data was obtained from annual surveys administered to each of the roughly ninety birthing facilities throughout the prefecture.

The Tottori Prefecture NHS Program

According to a September 2009 report on the status of NHS in Tottori, there was a gradual increase in the number of coordinated NHS programs in the prefecture from 2001 coinciding with the Japanese government’s funded programs at that time [21]. The 2009 report summarizes a five-year process of system improvement efforts which included the introduction of an annual report on the status of NHS in the prefecture. The following overview of NHS in Tottori Prefecture is based upon an analysis of these annual reports, available on the Prefecture’s website dating back to 2010 [22].

Detailed statistics of the annual screening rates are available from 2010 through 2020 with the first report in 2010 citing overall screening rates for the two years prior. The reported attainted screening rates are shown below in Table 4. From 2010, data are reported separately for children born under normal conditions and for those born in neonatal intensive care units (NICU). Since 2016, the overall screening rate has been above 99% and since 2017 the rate for children born in the NICU has been 100%.

**Table 4:** Screening rates in Tottori Prefecture from 2008 to 2020. NICU = Neonatal Intensive Care Unit

Year	Reported births non-NICU	Reported births NICU	Screened non-NICU	Screened NICU	Screening rate non-NICU	Screening rate NICU	Screening overall
2008	---	---	---	---	---	---	68.6%
2009	---	---	---	---	---	---	77.0%
2010	5,228	609	4,886	563	93.5%	92.5%	93.4%
2011	5,478	595	5,126	556	93.6%	93.5%	93.6%
2012	5,160	530	4,997	509	96.8%	96.0%	96.8%
2013	5,172	559	5,113	535	98.9%	95.7%	98.6%
2014	5,026	561	4,955	560	98.6%	99.8%	98.7%
2015	5,389	527	5,318	523	98.7%	99.2%	98.7%
2016	4,871	472	4,829	470	99.1%	99.6%	99.2%
2017	4,779	354	4,770	354	99.8%	100%	99.8%
2018	4,350	572	4,321	572	99.3%	100%	99.4%
2019	4,292	546	4,269	546	99.5%	100%	99.5%
2020	3,942	629	3,918	629	99.4%	100%	99.5%
Average	4881	541	4773	529	97.9%	97.8%	97.9%

NHS in Japan is conducted in each prefecture independently with little to no screening-related communication across prefectures. As such, it is difficult for information about children born outside of a prefecture of residence, as is the case in “Satogaeri” return home birth, to be obtained by healthcare and social welfare employees in the child’s home prefecture. This complicates not only precise monitoring of the NHS program but also timely follow-up and intervention in the case of children who receive a referral on their screening if they were able to receive a screening test at their place of birth. The Tottori Prefecture annual reports provide evidence of these difficulties. Table 5 demonstrates the difficulties in tracking the number of screenings per resident every year between 2010 and 2019, the years for which data was available at the time of this publication, showing a higher number of clinic births than the number of resident births reported by the prefecture. A 2009 Tottori Prefectural report states that approximately 10% of the annual reported births are from “Satogaeri” (return home births) or from births of residents from neighboring prefectures [21]. The difference in reported births and reported resident births, as shown in Table 5, suggests that an average of 21.4% of reported births are to “Satogari” return home mothers and neighboring prefecture mothers.

**Table 5:** Number of official resident births versus the number of births reported by screening clinics

Year	Reported births non-NICU	Reported births NICU	Total	Reported resident births	Difference	Percentage of possible “Satogaeri Bunben” births
2010	5,228	609	5,837	4,790	1,047	21.9%
2011	5,478	595	6,073	4,931	1,142	23.2%
2012	5,160	530	5,690	4,771	919	19.3%
2013	5,172	559	5,731	4,759	972	20.4%
2014	5,026	561	5,587	4,527	1,060	23.4%
2015	5,389	527	5,961	4,624	1,292	27.9%
2016	4,871	472	5,343	4,436	907	20.5%
2017	4,779	354	5,133	4,310	823	19.1%
2018	4,350	572	4,922	4,190	732	17.5%
2019	4,292	546	4,838	3,988	850	23.1%
Average	4,881	541	5,422	4,533	974	21.4%

In the Tottori Prefecture context, NHS tests are performed using either otoacoustic emission (OAE) testing or automatic auditory brainstem response (AABR) testing. The testing protocol stipulates children first be tested several days after birth while still in the hospital or clinic. In the event of a referral result on the first test, children are re-tested for confirmation approximately one month later when they return for a general checkup. If the confirmation check results in a second referral result, the child is referred to a designated ENT clinic for more precise diagnostic testing. NICU children, however, do not receive a confirmation test and instead are directly referred for more precise diagnostic testing. Tottori Prefecture’s annual reports include aggregated data for each step in this process. Because data are aggregated and thus not longitudinally connected throughout the screening, follow-up, and diagnostic process, and because of challenges presented by “Satogaeri” (return home birthing) and neighboring prefecture resident births, positive predictive value (PPV) calculations result in misleading figures. The figures in Table 6 demonstrate the challenges remaining for NHS programs of this nature in Japan.

**Table 6:** Initial and post-confirmation check referral rates. Note: Follow up test counts from 2016 to 2020 represent resident and non-resident numbers combined.

	Initial Refer		Confirm Refer	Refer for follow up		Follow up test			HI Diagnosed	
Year	Initial Refer	Initial Refer NICU	non-NICU only	“Normal”	(NICU)	“Normal”	(NICU)	“Normal”		(NICU)
2010	52	16	22	23	16	32	5		23	5
2011	59	10	31	36	10	34	3		19	2
2012	36	8	---	21	8	11	8		5	3
2013	60	11	26	30	9	28	3		17	3
2014	83	10	28	37	10	27	2		10	1
2015	66	10	21	38	10	38	4		22	4
2016	66	9	27	27	9	30	6		14	5
2017	87	9	32	33	9	37	4		13	4
2018	70	12	18	21	10	28	2		5	2
2019	58	7	19	32	6	33	6		11	5
2020	48	1	10	---	---	28	2		8	2
Average										

The data presented in Table 6 reveal an initial false positive rate, between the first referral and confirmation check, ranging from 37% to 62% with an average between 2010 and 2019 of 53%. Calculating a false positive rate from the follow up referral to follow up test and diagnosis reveals rates for “normal” children ranging from 0% to 76% with an average of 53% and rates for NICU children ranging from 17% to 90% with an average for the same time frame of 62%. For years when the number of confirmation tests is lower than the number of initial referrals, loss to follow may be a possible explanation, although not noted in annual reports. However, when referring for follow-up numbers exceed confirmed referrals or, likewise, when follow-up test administration numbers exceed refer for follow-up numbers, the reason for the increase is unexplained. It is the case in some years, as noted in the annual reports, that a portion of increases is due to referrals from outside the prefecture. In other cases, such as in 2017, the increase in numbers between the number of non-NICU children referred for follow-up, 33, and the actual number of follow up tests conducted, 37, is unexplained.

Overall, the annual summary data made publicly available by Tottori Prefecture is extraordinary, in terms of its detail, in the Japanese context. It reveals limitations within the current system but simultaneously evidences a gradually improving ongoing program.

The Shizuoka Prefecture NHS Program

Organized NHS in Shizuoka Prefecture began in 2004 when a governing investigative committee was formed. Initial efforts focused on ensuring that all birthing facilities were providing NHS testing. In 2005, the first-year data were aggregated and reported, 61.4% of birthing facilities in the prefecture were providing hearing screening services to newborns. From 2017 onwards, 100% of the birthing facilities in the prefecture have been providing NHS services.

In 2006, one of the authors began gathering NHS-related data from birthing facilities, hospitals, and clinics throughout the prefecture. In 2010, a staffed Infant Hearing Support Center was established, and data collection and management efforts were thereafter conducted by the center.

Results of NHS efforts throughout the prefecture have been reported annually every August since 2006 at the Shizuoka Prefecture Newborn Hearing Impairment Research Forum. The data reported upon at the forum are collected and compiled by the prefectural Infant Hearing Support Center which is housed in Shizuoka General Hospital and operated under management by the hospital’s Hearing and Language Support Center. From late March to early May of each year, a survey is compiled and sent to each of the birthing facilities in the prefecture. The survey gathers the following information:

· Name and contact information for the birthing facility

· Confirmation of birthing services for the specified year (yes/no)

· Newborn hearing screening test administration (yes/no)

· Testing equipment used (AABR, OAE)

· Testing equipment manufacturer information

· Average number of days after birth when tests are conducted (2-3, 4-5, 6 or more)

· Number of times test is administered to confirm result (1-2, 3-5, 6-9, 10 or more)

· Number of resident children tested for the year (question added in recent years)

· Number of non-resident children tested for the year (question added in recent years)

· Number of resident referrals for the year (question added in recent years)

· Name of the hospital to which referred children were directed

· Name of follow-up testing hospital if the response to the above is no

· Whether the birthing facility received details of the follow-up results (yes/no)

· If hearing screening results were recorded in the “mother/child” book (yes/no)

**Figure 1:**
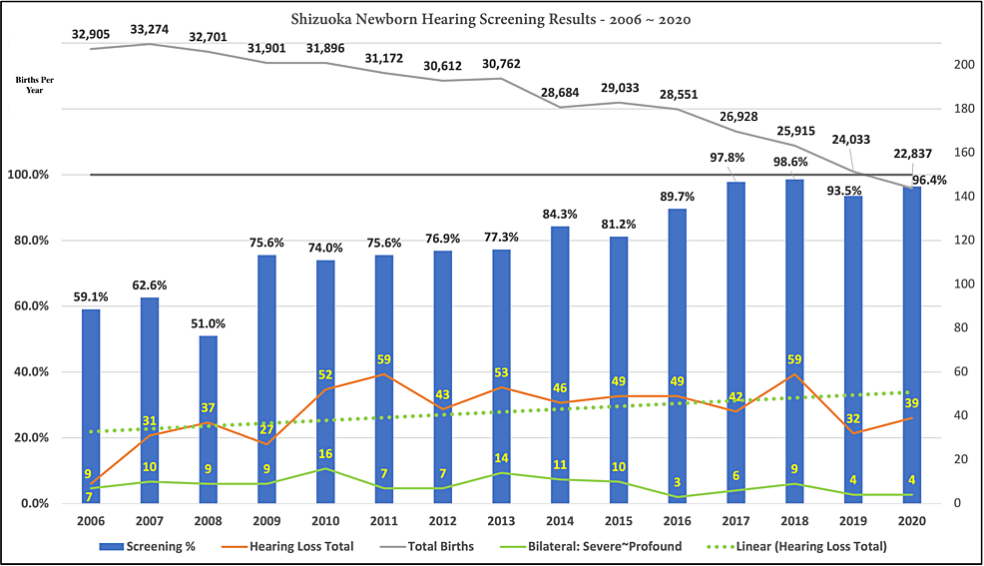
Newborn hearing screening rates per year from 2006 to 2020 including annual birth data and hearing impairment counts per year by category. Achieved screening rates are displayed as vertical bars with percentages displayed at the top of each bar for 2006 to 2020. The percentage scale for these figures is displayed on the left vertical axis. The total number of hearing impairment cases is plotted as an orange line from 2006 to 2020 with the scale displayed on the right vertical axis. The number of bilateral severe to profound hearing impairment cases is plotted as a green line from 2006 to 2020 using the same scale on the right vertical axis. The exact number of cases for total hearing loss and bilateral severe to profound hearing loss is displayed within the vertical screening rate bar with the total number on the top and the number of bilateral severe to profound cases on the bottom. The total number of births per year is displayed at the top of the figure.

From its inception in 2006 until 2020, surveys were printed and mailed to all birthing facilities in the prefecture. Responses were gathered via postal mail, fax, and, in isolated cases, via telephone by staff in the Hearing Support Center. The 2021 survey, collecting data from 2020, was conducted online using Google forms. A summary of the annual data from 2006 to 2020 is provided in Figure 1. In the figure, annual screening rates, are calculated using annual births reported by the prefecture. These annual births are plotted at the top of the graph displayed in the figure.

Here we argue several factors and epochs that influenced the years’ estimates. In the early years of compiling data on NHS in Shizuoka prefecture, screening rates were calculated based on the number of births reported by birthing facilities rather than on annual births reported by the prefecture. The practice of calculating screening rates from the number of reported births, from the annual survey, resulted in the reporting of higher screening rates than would result from using the total annual births. Additionally, the low percentages for the years 2006 through 2008 are a result of the difficulty in obtaining screening data from all birthing facilities because the task was being undertaken by one individual on a voluntary basis, as well as due to the difficulty in obtaining and correctly analyzing data for “Satogaeri” (return home) births and neighboring prefecture resident births. In 2009, a support staff member was hired to assist with the collection and aggregation of data. This accounts for the significant jump in the recorded screening rate between 2008 and 2009. Additionally, in 2016, a Prefectural financial assistance program was conducted to help hospitals and clinics purchase needed NHS equipment. This program, coupled with an NHS awareness campaign, are attributed as factors influencing the jump from an 89.7% screening rate in 2017 to 97.8% in 2018. In 2018, a shift was made from reporting data for the calendar year to reporting on a fiscal year schedule from April 1 to March 31. Simultaneously, the practice of reporting screening rates based upon births reported from birthing facilities was ceased. The slight decrease in reported screening rates from 2018 to 2019, 98.6% to 93.5%, is attributed, to an unknown degree, to administrative difficulty in shifting to a new aggregation schedule and method.

Births reported by birthing facilities, on the annual survey, are used to calculate referral rates, false positive rates, loss to follow percentages, and hearing impairment occurrence frequencies. The derived data are listed in Table 7.

**Table 7:** Shizuoka Prefecture newborn hearing screening data based upon birthing facility reported births. Moderate =40-70db, Severe = 70+db, SS = single sided hearing impairment, Freq = occurrence frequency

	Reported		Percentage rates			Hearing Impairment				
Year	Births	Test #s	Referral	False positive	Loss to follow	Moderate	Severe	SS	Total	Freq
2006	22,449	19,445	0.65%	48%	47.6%	2	7	-	9	0.03%
2007	27,900	20,841	0.76%	54%	36.5%	8	10	13	31	0.09%
2008	19,503	16,665	0.79%	61%	28.8%	8	9	20	37	0.11%
2009	25,970	24,116	0.90%	56%	61.5%	7	9	11	27	0.08%
2010	28,119	23,609	1.00%	56%	43.6%	11	16	25	52	0.16%
2011	29,713	23,556	1.12%	66%	28.8%	24	7	28	59	0.19%
2012	28,698	23,527	0.83%	58%	34.9%	11	7	25	43	0.14%
2013	28,609	23,772	0.76%	54%	17.2%	16	14	23	53	0.17%
2014	29,046	24,179	0.65%	58%	17.7%	15	11	20	46	0.16%
2015	28,424	23,574	0.75%	55%	16.4%	7	10	32	49	0.17%
2016	27,826	25,596	0.75%	55%	32.3%	10	3	36	49	0.17%
2017	27,405	26,324	0.63%	55%	27.5%	9	6	27	42	0.16%
2018	25,915	25,541	0.80%	52%	26.5%	16	9	34	59	0.23%
2019	23,067	22,481	1.35%	60%	59.7%	4	4	24	32	0.13%
2020	---	22,023	1.04%	77%	20.9%	7	4	28	39	0.17%
Average	26,617	23,018	0.85%	58%	33.3%	10.3	8.4	24.7	41.8	0.14%

The hearing impairment occurrence frequency, calculated from these data, ranges from 0.03% to 0.23% with an average of 0.14% over the 15-year span.

Nationwide screening status reports

The State of NHS in Japan, 2014-2019

As prefectures throughout Japan worked to provide sufficiently funded and organized NHS programs, the Ministry of Health, Labour, and Welfare began, in 2014, to publish national annual data summarizing the state of NHS in Japan [23]. These data are based on an annual survey sent to localities throughout the country. Information in the annual report is categorized as follows:

· Number of localities aware of newborn hearing screening status (aware/unaware)

· Of those aware, the number of facilities maintaining data

· Aggregated details from those maintaining data (births, screening #s, screening rate)

· Number of localities aware of test results 

· Of those aware of results, data from the subset who provided details 

· Number of localities aware of confirmation testing

· Of those aware of confirmation testing, data from the subset who provided details

· Number of localities aware of follow-up testing

· Of those aware of follow-up testing, data from the subset who provided details

· Number of localities that have implemented countermeasures for infants who did not receive initial screening testing

· Number of localities that have intervention assistance available for those identified with a need.

· Number of localities that have implemented public funding to assist with the cost of screening testing for both initial and confirmation tests

Five reports have been made publicly available from 2014 to 2019. No publication was made publicly available in 2015. The first annual report, from 2014, cites an NHS rate of 78.9%; however, this figure is calculated based on the number of births reported in the survey rather than on the total number of births in the country that year. Table 8 displays data aggregated from the five reports published between 2014 and 2019 together with nationwide birth counts obtained from the Ministry of Health, Labour, and Welfare statistics website [24].

**Table 8:** Data from the State of Newborn Hearing in Japan annual reports. HI = hearing impairment, reported births = births reported by the MHLW or on the newborn hearing screening survey, known screening rate = number of screenings divided by the total MHLW annual reported births

	Reported Births			Screening Rate		Referral	HI	HI
Year	MHLW	Survey	Screened	Reported	Known	%	Cases	%
2014	1,005,677	165,649	130,720	78.9%	13.0%	1.0%	195	0.149%
2016	976,978	375,449	310,917	82.8%	31.8%	1.1%	422	0.136%
2017	946,065	619,692	507,047	81.8%	53.6%	1.0%	593	0.117%
2018	918,400	779,459	677,709	86.9%	73.8%	1.2%	910	0.134%
2019	865,234	769,640	698,589	90.8%	80.7%	1.4%	1135	0.162%
Average								0.14%

The five-year average for hearing impairment occurrence frequency, calculated by averaging the five annual hearing impairment frequency rates, is at the same level as the average calculated from the 15-year dataset from Shizuoka Prefecture. Using this five-year average, estimating the expected number of hearing impairment cases not accounted for in this national summary report is possible. For example, in the most recent year, 2019, which shows the lowest gap between survey reported births and nationally recorded births (865,234 - 698,589 = 166,654 births), roughly 233 children (166,654 * 0.14%) can be estimated to have been born with some degree of hearing impairment. Although merely an estimate, when and whether children from this unaccounted-for group were identified and what intervention was conducted remains unknown. Additionally, the annual reports provide no information about hearing impairment diagnosis timing nor about intervention timing, details, or outcomes.

**Figure 2:**
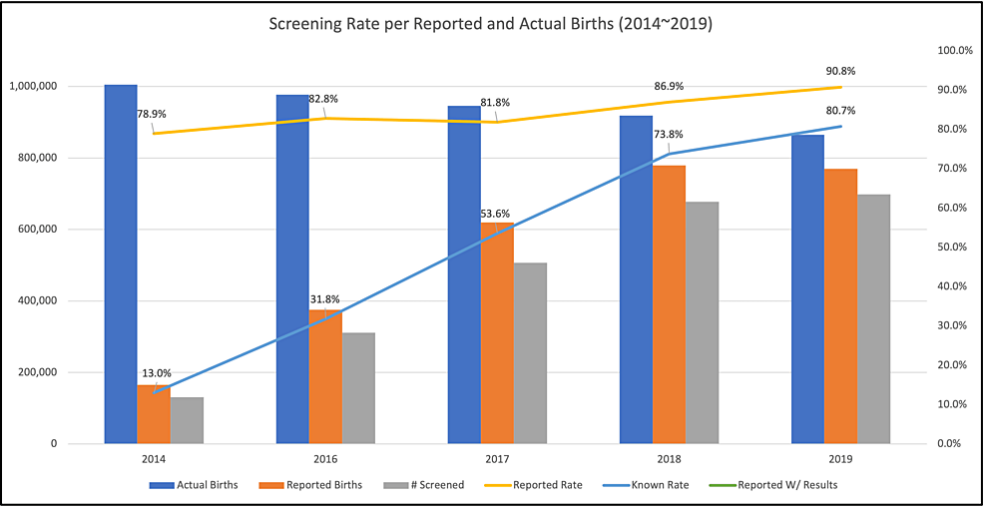
Actual births as reported annually by the Ministry of Health, Labour, and Welfare plotted together with births reported on the newborn hearing survey. The reported screening rate is from the newborn hearing survey. The known rate is calculated using annual total births instead of the total reported in the surveys. Actual births, displayed as blue vertical bars, are from the Ministry of Health, Labour, and Welfare e-Stat statistics website. Reported births, displayed as orange vertical bars, are the total numbers reported in the annual newborn hearing screening survey. The number of newborns screened, as reported from the annual survey, is displayed as a vertical grey bar. The scale for these vertical bars is displayed on the left vertical axis. Reported screening rates, from the annual survey, and the known rate calculated by dividing the total number of screenings by the number of annual births, are displayed as yellow and blue lines respectively. The percentage scale for these numbers is displayed on the right vertical axis.

The “known screening rate” conceptualization in Table 8 is utilized for the purpose of problematizing the screening rates cited in each annual report as these figures may be misconceived to represent the nationwide rates achieved in Japan. The difference between reported births, from localities, and reported screenings is not attributed to a concrete factor such as screening refusal. Additionally, the difference between the total births reported from the survey and the annual birth statistics is unaccounted for. As such, actual screening rates must fall in between the cited rate and the “known” rate. The two rates and their trends are visually represented in Figure 2.

Comparing data from the Shizuoka Prefecture NHS program with the data reported annually by the Ministry of Health, Labour, and Welfare resulted in the identification of a match in the average frequency rate for the occurrence of hearing impairment in newborns of 0.14%. The occurrence figure calculated for Okayama, 0.08%, is based upon a data set of 47,346 screening tests while the Shizuoka and national figures are calculated from significantly larger data sets. For Shizuoka, the figure is calculated based upon 345,267 screening test administrations over the course of fifteen years and for the nationally reported data on 2,324,982 screening test administrations over the course of five years. The occurrence frequency cited in the much smaller Tochigi Prefecture sample, 0.13% for the low-risk group, is very close to the figure cited for the Shizuoka and national data sets.

## Conclusions

The 2005 literature analysis conducted by Okubo et al. concluded “the effectiveness of UNHS in Japan is still equivocal…” based upon a review and analysis of 11 research articles, nine from which only screening accuracy information was obtained with the remaining two also providing information about screening effectiveness. This early review provides some evidence of the challenges NHS in Japan has faced since its inception in the early 2000s. Given the difficulties presented with tracking “Satogaeri” and other anomalous births in conjunction with a lack of a predetermined protocol for NHS programs, implementing, monitoring, and managing NHS programs in Japan remains a problematic challenge. In fact, all data-based summaries of NHS programs in Japan, surveyed for this review, are based upon annually collected and aggregated data. A digitized system, which provides real-time access to program metrics, would enable program improvement efforts that initiate a shift from retrospection to proactivity. As noted by Kamenov and Chadha in *Methodological quality of clinical guidelines for universal newborn hearing screening*, “despite unequivocal evidence on the advantages of early identification and intervention for hearing loss, very few countries have UNHS programs.” Nevertheless, efforts to make advancements in NHS implementation in Japan continue as the Ministry of Health, Labour, and Welfare recently convened a yearlong investigative commission for the creation of a basic policy for the promotion of early detection and early rehabilitation of children with hearing loss. Additionally, in the Shizuoka Prefecture context, a plan referenced above, to digitize the record-keeping portion of the NHS program for the purpose of streamlining implementation and enabling a protocol to be clearly defined and monitored is underway. Such efforts, coupled with careful consideration of successful existing models in the world, will result in further progress in implementing effective NHS programs in Japan.

None of the information obtained for this article included reference to established protocol in terms of expected referral rates, false positive rates, sensitivity, positive predictive value, or other quality control standards. In regard to referral rates, for the data available, figures fall well within the target of less than 4% recommended by the JCIH. While still an optional screening test, NHS in Japan is evidenced, by the data available, to have significantly expanded over the past twenty years. The data analyzed for this article indicate a hearing impairment occurrence frequency for newborns in Japan of roughly 0.14% or one of every 1,400 births. From a universal NHS program perspective, screening rates have advanced but still fall short of the recommended 95% or higher target set by the JCIH. Additionally, the figures cited represent a percentage of a subset of births rather than of the entire annual newborn population. Calculating a known screening rate will provide a more accurate representation of the status of NHS and thus enable more effective improvement efforts. Also, the establishment of an NHS program protocol will enable subsequent program monitoring, analysis, and improvement. A protocol of this nature may include targets such as

a. Target screening rate of 95% or higher by one month of age

b. Referral rate for well babies of 4% or lower although current achieved rates suggest a target of 1.5% as being realistic (data for establishing a NICU referral target was unavailable, however, a higher target (7%-8%) may be realistic

c. Follow-up testing within four weeks of birth for 90% or more of referred cases

d. Monitoring of positive and negative predictive value for the purpose of test equipment maintenance, methodology quality control, and to ensure effective early intervention

e. Monitoring of intervention commencement timing to ensure timely early intervention

Plans to establish, implement, and manage such a protocol are a component of the evolving Shizuoka Prefecture program introduced in this review. Future research focusing on NHS program implementation in Japan should utilize a clearly defined and agreed-upon protocol of this nature.
